# Extracellular Vesicles in the Development of the Non-Alcoholic Fatty Liver Disease: An Update

**DOI:** 10.3390/biom10111494

**Published:** 2020-10-30

**Authors:** Vicneswarry Dorairaj, Siti Aishah Sulaiman, Nadiah Abu, Nor Azian Abdul Murad

**Affiliations:** UKM Medical Molecular Biology Institute (UMBI), Universiti Kebangsaan Malaysia (UKM), Jalan Yaacob Latiff, Cheras, Kuala Lumpur 56000, Malaysia; vicneswarry@yahoo.co.uk (V.D.); nadiah.abu@ppukm.ukm.edu.my (N.A.); nor_azian@ppukm.ukm.edu.my (N.A.A.M.)

**Keywords:** exosome, microvesicles, apoptotic bodies, adipocyte, hepatocyte, cell to cell communication, NAFLD, insulin resistance, fibrosis

## Abstract

Non-alcoholic fatty liver disease (NAFLD) is a broad spectrum of liver damage disease from a simple fatty liver (steatosis) to more severe liver conditions such as non-alcoholic steatohepatitis (NASH), fibrosis, and cirrhosis. Extracellular vesicles (EVs) are a heterogeneous group of small membrane vesicles released by various cells in normal or diseased conditions. The EVs carry bioactive components in their cargos and can mediate the metabolic changes in recipient cells. In the context of NAFLD, EVs derived from adipocytes are implicated in the development of whole-body insulin resistance (IR), the hepatic IR, and fatty liver (steatosis). Excessive fatty acid accumulation is toxic to the hepatocytes, and this lipotoxicity can induce the release of EVs (hepatocyte-EVs), which can mediate the progression of fibrosis via the activation of nearby macrophages and hepatic stellate cells (HSCs). In this review, we summarized the recent findings of adipocyte- and hepatocyte-EVs on NAFLD disease development and progression. We also discussed previous studies on mesenchymal stem cell (MSC) EVs that have garnered attention due to their effects on preventing liver fibrosis and increasing liver regeneration and proliferation.

## 1. Introduction

Non-alcoholic fatty liver disease (NAFLD) is a common cause of chronic liver disease worldwide [1]. Currently, the prevalence of NAFLD in the general population is about 20–30% in Western countries and about 5–18% in Asian countries [1,2,3]. This NAFLD prevalence is expected to increase over time due to the increasing prevalence of obesity worldwide [1]. According to World Gastroenterology Organization, NAFLD is defined by having excessive triglycerides accumulation in the liver (known as steatosis), with the absence of significant alcohol consumption, hypothyroidism, or drug abuse [4,5]. About 3–5% of individuals with fatty liver can develop non-alcoholic steatohepatitis (NASH), which is characterized by hepatic inflammation and injury (ballooning of the liver) [4]. The progression to NASH is a serious condition, as the individuals with NASH have higher risks of developing cirrhosis and hepatocellular carcinoma (HCC) [5]. Therefore, NAFLD represents a broad spectrum of liver damage ranging from steatosis to non-alcoholic steatohepatitis (NASH) and cirrhosis, resulting in liver failure and HCC [4].

The exact mechanisms leading to NAFLD and NASH are unknown. Several hypotheses are known, and insulin resistance seems to be the center of this disease pathogenesis [6,7,8]. The NAFLD pathogenesis follows the “three-hit” hypothesis involving steatosis, lipotoxicity, and inflammation [4,8]. The first hit addresses the influence of whole-body insulin resistance (IR) that causes an excessive accumulation of triglycerides in the liver (steatosis) [4]. Typically, most fatty acids (FA) are stored in the adipose tissue as triacylglycerol (TAG). However, the presence of IR and metabolic changes in obese individuals cause the re-routing of TAG storage to skeletal and liver tissues [4,8]. Following that, steatosis increases the signaling and production of pro-inflammatory mediators, in which additional macrophages are recruited from the blood mononuclear compartment to the inflammation foci, as seen in NASH individuals [4]. Excessive fat accumulation and liver inflammation are toxic to hepatocytes (lipotoxicity). This lipotoxicity effect could lead to severe organelle dysfunctions, particularly mitochondrial dysfunction and endoplasmic reticulum stress [4,8]. The increased production of reactive oxygen species (ROS) eventually leads to hepatocyte death [4,8]. This vicious cycle of steatosis, lipotoxicity, and inflammation causes hepatocyte deaths and the activation of hepatic stellate cells (HSCs), leading to fibrosis formation and more severe liver outcomes that eventually destroy the liver organ [4,8].

Recent evidence indicates that extracellular vesicles (EVs) have important roles in the NAFLD pathogenesis. One example is the EVs derived from the adipose tissue (adipocyte-EVs) [9]. Adipocytes secrete various adipokines such as adiponectin, leptin, visfatin, resistin, and adipsin, as mediators to regulate other organs [9,10]. Adipocyte-EVs are distinctly different from these known adipokines, but they could modulate specific recipient cells’ changes based on their cargos [9]. Thus, EVs can serve as signals for long-distance cell-to-cell communications with specific deliverable bio-active components. Another example is the EVs released from damaged hepatocytes (hepatocyte-EVs), in which these hepatocyte-EVs contribute to the progression of liver damage by activating the liver non-parenchymal cells such as liver sinusoidal epithelial cells (LSEC) and HSCs [11,12]. Given the potential involvement of EVs in NAFLD pathogenesis, in this review article, we will summarize the recent findings from previous studies of EVs and focus on their role in NAFLD pathogenesis. We will also discuss the potential of these EVs as an option for liver treatment.

## 2. Extracellular Vesicles (EVs): Biogenesis and Classes

EVs are small membrane vesicles released from the cells in a highly regulated manner and modulate the expressions of the genes and pathways in target cells [13,14]. EVs are released from almost all cells and exist in many tissues and biological fluids, including blood and urine [13]. Their bioactive cargos contain various components, such as proteins, lipids, and nucleic acids (DNA, RNA, microRNA, and long noncoding RNA (lncRNA)) [13]. More significant releases of EVs are observed in pathological conditions such as infection, cancer, and metabolic diseases [9,15,16,17]. Based on their sizes and biogenesis, EVs are mainly divided into exosomes, microvesicles (MVs), and apoptotic bodies [13].

### 2.1. Extracellular Vesicles (EVs): Exosomes

**Figure 1:**
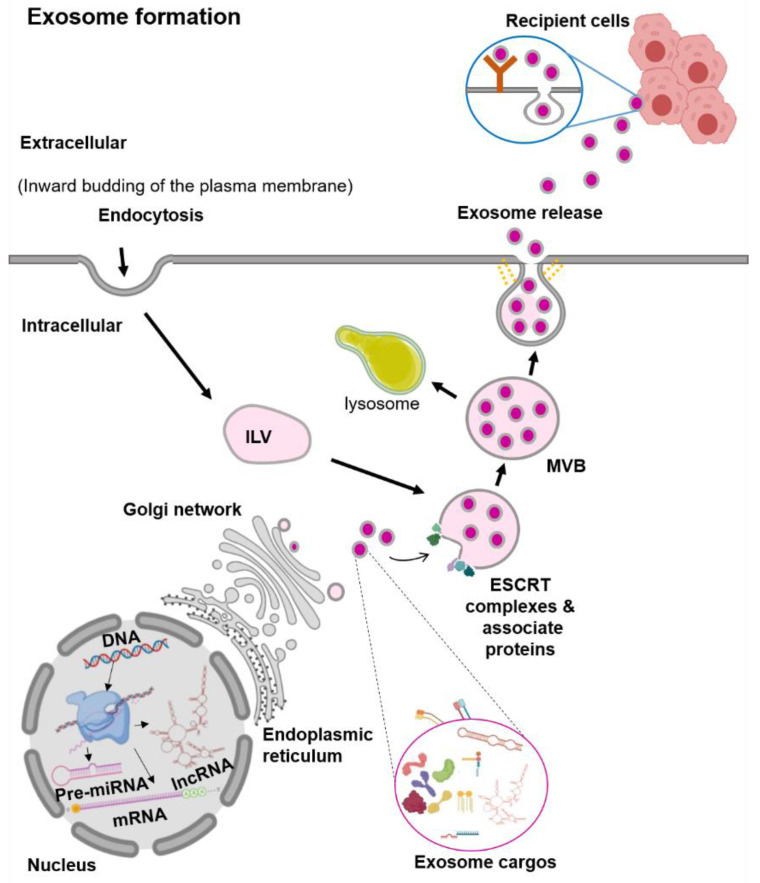
Schematic representation of the exosome biogenesis. Abbreviation: endosomal sorting complex required for transport (ESCRT), intraluminal vesicles (ILV), multivesicular bodies (MVBs).

Exosomes are the smallest EVs, with a size range from 30 to 100 nm in diameter [13]. They are generated within the endosomal network, during the sorting of various intraluminal vesicles (ILV) for recycling, degradation, or exocytosis (Figure 1) [13,14]. The first step in the exosome biogenesis is early endosomes’ formation by the inward budding of the plasma membrane [13,14]. Then, these early endosomes are merged with the cytoplasmic contents to form ILV. Several ILVs containing different cargos are known as multivesicular bodies (MVBs). In the MVBs, the ILVs containing cargos for degradation are delivered to lysosomes. In contrast, ILVs contents destined for exocytosis in the late endosomes further undergo a series of transformations to fuse with the plasma membrane and are then released out [13,14]. Exosome vesicle formation requires the Endosomal Sorting Complex Required for Transport (ESCRT) [14]. There are four ESCRT complexes (ESCRT-0, ESCRT-I, ESCRT-II, and ESCRT-III) and their associated proteins (VPS4, TSG101, and ALIX) [18]. In vitro studies showed that the ESCRT-0 protein sorts the ubiquitinated protein cargos, while the ESCRT-I and ESCRT-II proteins induce the membrane budding [18]. The recruitment of the ESCRT-III protein to the site of ESCRT-I and -II is through the ALIX protein, in which ALIX binds to TSG101 (a component of the ESCRT-I complex) and CHMP4 (a component of ESCRT-III) [19]. Finally, to complete the budding, VSP4 is recruited to the ESCRT-III complex and drives the vesicle neck scission and dissociation [18].

There is evidence of the exosome vesicle formation that can be independent of the ESCRT complexes, in which the process involves lipids and tetraspanin [20]. For RNA molecules, the sorting of these RNAs into the exosome seems to be lipid-mediated [21]. The specific sequences in the RNAs enhance the affinity of RNAs to bind to the lipid bilayer. This RNA–lipid binding depends on several factors such as sphingosine concentration, lipid raft or structure, and the hydrophobic modifications [21]. The presence of specific lipids such as ceramide, lysophospholipids, and glycosphingolipids on the plasma membrane induces spontaneous bending inward to produce ILV [22]. The ceramidase and sphingosine kinase enzymes convert the ceramide to sphingosine and sphingosine-1-phosphate (S1P). This activation of the S1P receptor on the plasma membrane induces the reorganization of the membrane proteins, including CD9 and CD63, into specialized units of tetraspanin regions or known as the Tetraspanin-Enriched Microdomains (TEMs) [23,24]. These TEMs form MVBs clusters by interacting with various transmembrane and signaling proteins [25]. Although the formation of vesicles can happen without the ESCRT complexes, the process of exosome protein cargo sorting into ILVs is impaired [25]. Therefore, these findings suggest that the biogenesis of exosomes is a tightly coordinated process involving both ESCRT-dependent and -independent pathways.

### 2.2. Extracellular Vesicles (EVs): Microvesicles

**Figure 2:**
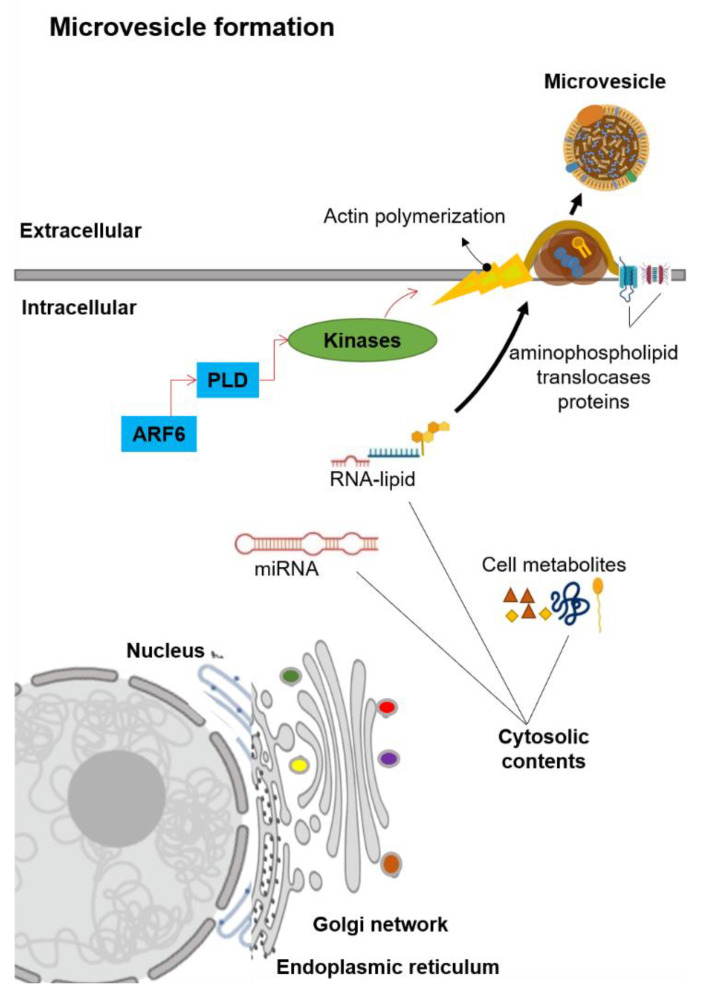
Schematic representation of the microvesicle biogenesis. Abbreviation: ADP-ribosylation factor-6 (ARF6), microRNA (miRNA), phospholipase D (PLD).

Microvesicles are generally in the size range of 100–1000 nm in diameter. Unlike exosomes, MVs are generated by the plasma membrane’s outward budding into the extracellular space (Figure 2) [13]. The MVs formation is first mediated by the redistribution of phospholipids within the plasma membrane [26]. This redistribution of phospholipids is regulated by the aminophospholipid translocases (the proteins that transfer phospholipids from one part of the plasma membrane to another) [26,27]. Two main groups of aminophospholipid translocases proteins are the flippases that transfer phospholipids from the outer extracellular-facing region into the inner cytoplasmic-facing region and the floppases that transfer phospholipids from the inner cytoplasmic-facing region to the outer extracellular-facing regions [26,27]. The translocation of phosphatidylserine initiates the budding formation to the outer extracellular-facing region of the membrane [28]. A signaling cascade is started by the ADP-ribosylation factor 6 (ARF6), in which it activates the phospholipase D (PLD, an enzyme that synthesizes phosphatidylserine via two-step reactions) [29,30]. This activation of PLD recruits the kinases to the plasma membrane, and these kinases start the actin polymerization [30]. The ERK kinase enzyme phosphorylates myosin light-chain kinase, which in turn phosphorylates the myosin light chain to trigger the release of MVs [13,30]. Notably, this signaling cascade did not influence exosomes secretion [31], which suggests that the biogenesis of MVs is distinct from the exosomes.

### 2.3. Extracellular Vesicles (EVs): Apoptotic Bodies

**Figure 3:**
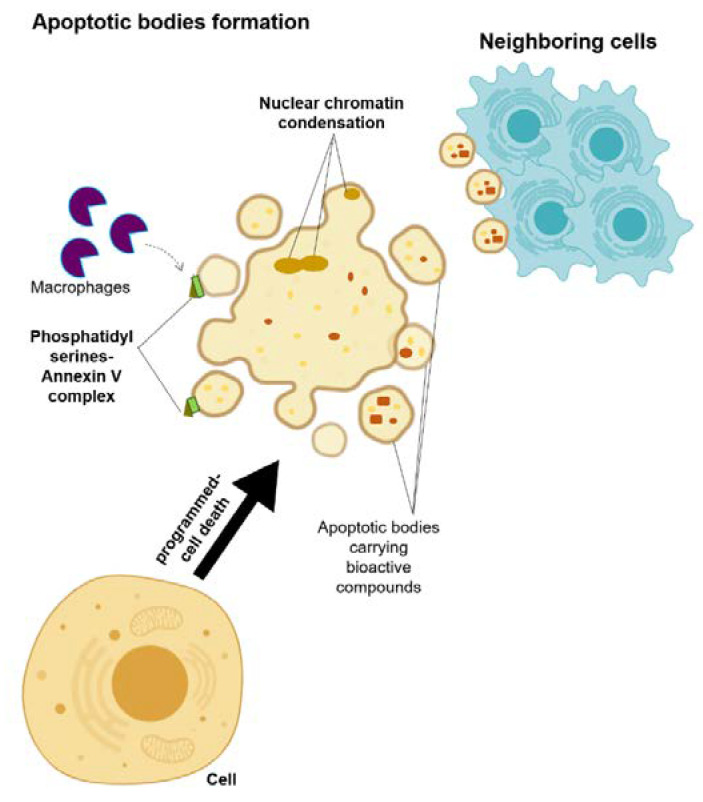
Schematic representation of the microvesicle biogenesis.

Apoptotic bodies are membrane-enclosed vesicles released from dying cells that underwent apoptosis (Figure 3) [13,32]. The size of apoptosomes is diverse, with the size ranging from big vesicles (500–400 nm) that are often seen with organelles inside to small vesicles (50–500 nm) [13,32]. A cell that activates the programmed-cell death undergoes a series of processes, including nuclear chromatin condensation, membrane blebbing, and disintegration of the cellular components into apoptotic bodies [32]. Most of the time, these apoptotic bodies are taken in by the macrophages due to the translocation of the phosphatidylserines on the outer side of the apoptotic bodies’ membrane, which can bind to the Annexin V that is recognized by the macrophages [32,33]. Despite the distinct biogenesis and mechanisms for each of these EVs, their ability to carry bioactive cargos and transfer them to the receiving cells is a common trait of these EVs [13]. Therefore, their roles in the development of the disease are considered as a new hypothesis, and hence, they may answer the underlying mechanism of disease development and progression, as well as allowing for the future development of a new treatment approach.

## 3. Extracellular Vesicles (EVs): Adipocyte-Derived EVs in NAFLD

**Figure 4:**
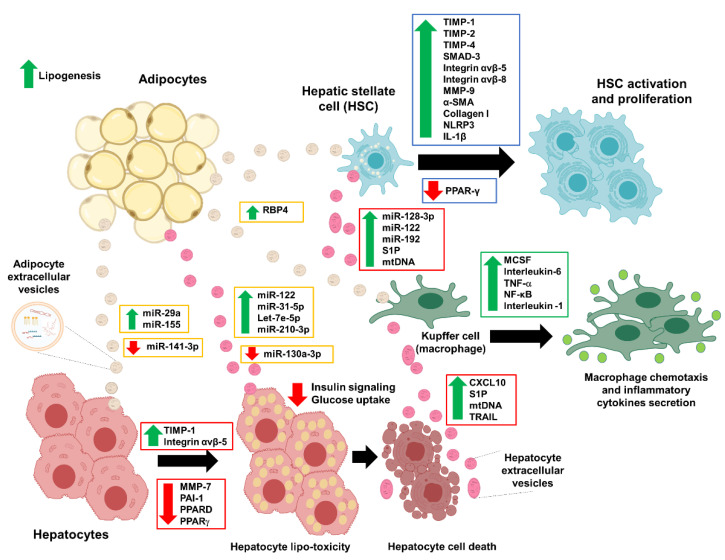
Illustration of the extracellular vesicles’ involvement in NAFLD development. Adipocyte-derived extracellular vesicles mediate the endocrine effects in hepatocytes, hepatic stellate cells, and macrophages (Kuffer cells) in the early phase of the NAFLD development. The disease progression becomes worse when the hepatocytes release their extracellular vesicles that promote the formation of fibrosis. The green arrow represents upregulation, and the red arrow represents downregulation. Abbreviation: α-smooth muscle actin (α-SMA), C-X-C-motif chemokine 10 (CXCL10), interleukin-1 β (IL-1β), macrophage colony-stimulating factor (MCSF), matrix metalloproteinase-7 (MMP-7), matrix metalloproteinase-9 (MMP-9), mitochondrial DNA (mtDNA), non-alcoholic fatty liver disease (NAFLD), nuclear factor kappa-light-chain-enhancer of activated B cells (NF-κB), NLR family pyrin domain containing 3 (NLRP3), peroxisome proliferator-activated receptor delta (PPARD), peroxisome proliferator-activated receptor gamma (PPAR-γ), plasminogen activator inhibitor-1 (PAI-1), sphingosine-1-phosphate (S1P), tissue inhibitor of matrix metalloproteinase-1 (TIMP-1), tissue inhibitor of matrix metalloproteinase-2 (TIMP-2), tissue inhibitor of matrix metalloproteinase-4 (TIMP-4), TNF-related apoptosis-inducing ligand (TRAIL), and tumor necrosis factor-alpha (TNF-α).

In response to various stimuli, EVs could be released by adipocytes as adipokines to modulate endocrine changes in the near or distant cells (Figure 4). Although it is unclear whether these EVs can genuinely act as specialized cargo or adipokines, previous studies have shown that adipocyte-derived EVs (adipocyte-EVs) did have functional outcomes in regulating metabolic and endocrine organs (Table 1) [34,35,36,37,38]. The first study that identified the role of adipocyte-EVs in insulin resistance was performed in a mice model of obesity [34]. In this study [34], adipocytes-EVs were isolated from the leptin-deficient obese mice and high-fat diet mice, in which these adipocyte-EVs were cultured into the primary macrophage cells for 14 days. Adipocyte-EVs from the obese and high-fat-fed mice models activated these macrophages and increased the production of macrophage colony-stimulating factor (MCSF), interleukin-6 (IL-6), and tumor necrosis factor-alpha (TNF-α) [34]. Moreover, in the same study, culture media from these activated macrophages caused impaired glucose uptake and insulin resistance in myocytes [34]. Another study isolated EVs from the adipose tissue macrophages (ATMs) of the obese mice and injected them into the lean mice [38]. This treatment of obese EVs caused impaired glucose tolerance and insulin sensitivity in lean mice [38]. In contrast, the treatment of EVs from lean ATMs to the obese mice improved the insulin sensitivity and normalized glucose levels [38]. Notably, the EVs derived from obese ATMs also induced hepatic and muscle insulin resistance (IR) together with evidence of lipid dysregulation, and this IR effect is partly due to the miR-155 that was enriched in the EVs of obese ATMs [38]. Another study of these obese ATMs also found similar IR effects with miR-29a enrichment in EVs of the obese ATMs. This miR-29a induces IR via its target gene, peroxisome proliferator-activated receptor delta (*PPARD*) [39]. A study of the obese individuals also confirmed that obese adipocytes-EVs contain various microRNAs, and these microRNAs target genes involved in the inflammatory and fibrotic signaling pathways, including the transforming growth factor-beta (TGF-β) and Wnt/β-catenin signaling [36].

Another example of the adipocyte-EVs that mediate the endocrine effects is the study of women with gestational diabetes mellitus (GDM), in which the adipocyte-EVs isolated from their primary omental adipose tissues were positively correlated with the fetal growth [35]. In these GDM adipocyte-EVs, there was an enrichment of the proteins involved in sirtuin signaling, oxidative phosphorylation, rapamycin signaling pathways, and the upregulation of the genes involved in glycolysis and gluconeogenesis [35]. Notably, the co-culture of obesity-derived adipocyte-EVs with the hepatocytes showed that adipocyte-EVs are taken into the hepatocytes and HSCs [37]. In this study [37], after 48 h of exposure to obese adipocyte-EVs, there were dysregulations of the TGF-β signaling molecules such as the increased expressions of tissue inhibitor of matrix metalloproteinase-1 (TIMP-1) and integrin ανβ-5, and the reduced expressions of matrix metalloproteinase-7 (MMP-7) and plasminogen activator inhibitor-1 (PAI-1) [37]. In HSCs, the obese adipocytes-EVs induced expressions of TIMP-1, TIMP-4, SMAD-3, integrins ανβ-5 and -8, and MMP-9, indicating the early formation of fibrosis [37]. The effects of adipocyte-EVs on hepatocytes are partly due to the microRNAs residing in the adipocyte-EVs cargo. One example is miR-141-3p, which was significantly reduced in obese adipocyte-EVs [40]. MiR-141-3p is responsible for the normal phosphorylation of AKT upon the insulin stimulation; thus, the low level of miR-141-3p may explain the impaired insulin signaling and glucose uptake in the hepatocytes [40]. These previous findings suggest that adipocyte-EVs may influence other cell types based on the status of the adipocytes. Hence, the development of hepatic IR in NAFLD may be partly explained by these adipocyte-EVs derived from IR or metabolic-changed adipocytes.

Circulating adipocyte-EVs may also be involved in the whole-body metabolic status (distant signaling). In a study of the obese individuals that underwent gastric bypass surgery, the circulating adipocyte-EVs, mainly their microRNAs cargo after the surgery, were associated with improved insulin signaling when compared to their adipocyte-EVs microRNAs cargo before the surgery [41]. In this study [41], the circulating adipocyte-EVs were confirmed by the adipocyte marker of FABP-4. Another study of circulating adipocyte-EVs is from the 1012 patients with vascular disease, in which the plasma adipocyte-EVs from vascular disease individuals had a high level of cystatin-C, and this adipocyte-EVs–cystatin-C level was associated with the 57% risk of having metabolic syndrome [42]. In the mice model of obesity, the aerobic training reduced the expression of miR-122, miR-192, and miR-22 in the EVs isolated from their serum samples [43]. This reduced expression of miR-22 was negatively correlated with adipogenesis and insulin sensitivity markers in adipocytes. Liver expression of PPARγ was also negatively correlated with miR-122 expression in the serum level. Due to aerobic training, obesity-induced steatohepatitis was prevented, and this may be explained by the changes seen in the serum EVs of microRNAs [43]. However, in this study [43], the circulating serum EVs were not characterized by their origins; thus, it is difficult to confirm the microRNAs roles. To ensure that these circulating adipocyte-EVs can manifest similar effects as adipocyte-EVs derived from the adipose tissues, a study was performed in the obese mice induced by genetic modification [44]. In this study [44], plasma adipocyte-EVs from the genetically engineered obese mice (confirmed by perilipin A marker) were injected into normal wild-type mice. These circulating adipocyte-EVs caused the accumulations of the inflammatory monocytes in the blood and the adipose tissues of the normal mice [44]. In the plasma samples of the NASH mice model, there are significant enrichments of EVs derived from different types of cells [45]. In this study [45], the hepatocyte-EVs are enriched as early as at 10 weeks of high-fat feeding compared with the inflammatory EVs derived from the macrophages, and these hepatocyte-EVs are significantly correlated with NASH development. However, due to the limited findings available, the interpretation of these data should be viewed with caution. Moreover, one of the problems with circulating adipocyte-EVs studies is the specific marker used to identify these EVs in circulating biological fluids [46]. As discussed above, adipocytes markers such as adiponectin, FABP-4, perilipin A, and RBP-4 have been used for adipocyte-EV-specific markers [41,44]. However, it is also shown that adiponectin in the exosomes only accounts for a small proportion of the total secreted adiponectin [47], and this adiponectin may not be a reliable marker for adipocyte-EVs. As for perilipin A, the EVs with perilipin A-positive were greater in high-fat-induced obesity in the mice and humans with metabolic syndrome [44]. Therefore, perilipin A is not necessarily specific for adipocytes. Nevertheless, the development of IR is the hallmark of early NAFLD development, and it may indeed be partly due to the communications between adipocytes and the hepatocytes via the EVs. Further studies to evaluate the cargos of these adipocyte-EVs may uncover the molecular signals of the IR.

## 4. Extracellular Vesicles (EVs): Damaged Hepatocytes Roles in NAFLD

The accumulations of the lipids lead to lipotoxicity in the hepatocytes and eventually lead to hepatocyte damage and cell death [6,60]. Previous studies have shown that excessive lipids stimulate the release of hepatocyte-EVs with pro-inflammatory molecules inside (Figure 4 and Table 1) [11,48]. These pro-inflammatory hepatocyte-EVs may promote fatty liver progression to NASH and fibrosis via the stimulation or activation of nearby liver cells such as Kupffer cells and HSCs [60]. In a study of hepatocyte-EVs due to lipotoxicity, the C-X-C motif chemokine 10 (CXCL10) protein was enriched in the hepatocyte-EVs cargo and induced the recipient Kuffer cells chemotaxis [54], which is the activated Kupffer cells releasing various pro-inflammatory cytokines such as TNF-α and interleukins (IL-1 and IL-6) [49], as well as the inflammasomes such as NLR family pyrin domain containing 3 (NLRP3) and ASC [58]. Similarly, another study of hepatocyte-EVs on macrophages showed that these hepatocyte-EVs have higher pro-inflammatory lipids, such as S1P [48] that also can induce macrophage chemotaxis. This release of hepatocyte-EVs is partly due to ER-stress mediated by inositol-requiring enzyme 1α (IRE1α) signaling [61]. Again, in these ER-stress mediated hepatocyte-EVs, there are enrichments of ceramide metabolite of S1P [61]. In plasma samples of NASH mice models, the isolated hepatocyte-EVs have higher mitochondrial DNA (mtDNA) and intact mitochondria, which can activate the TLR9 ligands [62]. The activation of TLR9 will result in the downstream activation of NF-κB-dependent pro-inflammatory cytokines in macrophages [63]. A similar observation was seen in the study of microvesicles released from the lipotoxic hepatocytes [64]. Unlike the exosomes, the internalization process of these hepatocyte-microvesicles is the trigger of the pro-inflammatory pathway activation in the macrophages [64]. Importantly, a recent publication showed that adipocytes could also take these lipotoxic hepatocyte-EVs and result in adipocyte remodeling to increase the fat accumulation and expression of lipogenesis genes [56]. In this study [56], the authors showed that hepatocyte-EVs have enrichments of miR-122, let-7e-5p, miR-31-5p, and miR-210-3p in their cargos, and these hepatocytes-EVs were taken in by the adipocytes, not myocytes, thus indicating the feedback interaction between the lipotoxic liver and adipose tissues. Interestingly, the treatment of hepatocytes-EVs with an enrichment of miR-130a-3p level to the adipocytes improved the glucose uptake and reduced the lipid droplets in the adipocytes [50]. The improvements were due to the suppression of the miR-130a-3p target gene, *PHLPP2,* and its downstream pathway of the AKT-AS160–GLUT4 signaling [50].

In addition to macrophages, hepatocyte-EVs can induce activation of the HSCs, which is the hallmark of fibrosis formation. In a study of high-fat diet mice, hepatocyte-EVs microRNA, miR-128-3p suppressed the *PPAR-γ* expression in HSCs [53]. This reduction of *PPAR-γ* expression led to HSCs activation with the increase of profibrogenic genes expressions such as α-smooth muscle actin (α-SMA), collagen-I, and TIMP-2; HSC proliferation; and wound-healing responses [53]. This finding is replicated again in other studies, in which the lipotoxic hepatocyte-EVs increased the expression of pro-fibrotic markers of *TGFβ-1*, *CTGF*, *COL1A1*, and *α-SMA* in HSCs [51,57]. Intriguingly, within these hepatocyte-EVs, different microRNAs are identified as the molecule of interest, despite having the same downstream effects in HSCs. Thus, there are likely more molecular mediators in hepatocyte-EVs that can induce inflammation and fibrosis in NAFLD. Therefore, more studies are needed to explore these molecules. Intriguingly, the treatment of Ras homologous (Rho)-associated coiled-coil-containing protein kinase 1 (ROCK1) inhibitor (fasudil) reduced lipotoxicity induced by hepatocyte-EVs via the reduction of DR5 signaling and ROCK1 [11]. In this study [11], the hepatocyte-EVs contain the TRAIL protein, which is a known driver for the apoptotic signaling cascade. These TRAIL proteins bind to its DR5 receptor on the recipient macrophages to activate the macrophage chemotaxis [11]. Even though no information is available for how these hepatocyte-EVs are internalized, one study managed to show that a surface protein ectoenzyme Vanin-1 (VNN1) is required for the process, especially for the recipient endothelial cells, and this internalization of the hepatocyte-EVs leads to angiogenesis [65]. Although the evidence is limited, there is a consistency of evidence showing that hepatocyte-EVs can stimulate the macrophages and HSCs activation and thus contribute to the worsening of the liver conditions.

## 5. EVs from the Mesenchymal Stem Cells as a Treatment Option

There are significant interests in the mesenchymal stem cells (MSCs) and their EVs, regarding whether these MSC EVs could regenerate or restore the organ condition (Table 2). One of the early reports is the investigation of the EVs cargos from the adipose-derived stem cells (ADSCs) and discovered that the EVs contained sets of microRNAs to suppress target genes in the recipient cells [66]. Similarly, another study showed that the EVs from ADSCs have a distinct profile of proteins in their cargos, and these cargos are different for each healthy individual [67]. In contrast, in a study of obese individuals, the ADSC EVs were not different in terms of the numbers and size between obese and non-obese individuals, although there was a reduction of miR-126 in the EVs of obese individuals [68]. This reduction of miR-126 in the obese EVs caused impaired angiogenesis in the endothelial cells upon being treated with the obese EVs [68]. Interestingly, the treatment of platelet-derived growth factor (PDGF) on the ADSC caused EVs’ release with greater angiogenesis effects on the endothelial cells [69]. This study [69] suggests that the external stimulations can change the effects or properties of the secreted EVs, and depending on the objective, these EVs can be used as a new treatment approach.

The potential of EVs as a treatment is supported by a study of mice ADSCs in which the ADSC EVs were isolated and used to inhibit the proliferation and migration of the vascular smooth muscle cells (VSMCs) together with the reduction of macrophages recruitment and inflammatory cytokines, thus reducing the intimal hyperplasia [70]. In another study of ADSCs in pig, the ADSC EVs were isolated and injected into the renal artery of the high-fat diets pigs for four weeks. This treatment of ADSC EVs protected the renal structure and function despite the high-fat diets [71]. These renal protective effects are partly due to IL-10 presence in the cargo of ADSC EVs, as the inhibition of IL-10 in ADSC and their IL-10-deficient-EVs abolished the renal protective effects [71]. Interestingly, in the mice model of obesity, treatment of ADSC EVs improved insulin sensitivity, reduced obesity, and alleviated hepatic steatosis in the mice [72]. These improvements were partly due to the activation of anti-inflammatory phenotypes in M2 macrophages via the trans-activation of arginase-1 by ADSC EVs enriched-STAT3 molecules [72].

A few studies reported that EVs could improve liver conditions. A study of carbon tetrachloride (CCl4)-induced liver injury in the mice showed that EVs derived from the human umbilical cord-MSCs reduced the fibrosis progression as well as lessened the inflammation and collagen deposition in the liver, partly via the inactivation of the TGF-β1/SMAD signaling [73]. In another hepatic injury study, the EVs from MSC exhibited protective effects on the hepatocytes despite being treated with liver injury stimulant drugs, partly via the upregulation of hepatocyte proliferation [74]. Other studies of EVs from the various origins of MSCs showed similar hepatic protective and regenerative effects, as seen in the bone-marrow-derived MSCs [75,76,77,78,79,80], human umbilical cord-derived MSCs [81,82,83], human liver stem cells [84,85], induced pluripotent stem cell-derived MSCs [86], human embryonic-derived MSCs [64], human menstrual blood-derived MSCs [87], and adipose-derived MSCs [88,89,90,91].

As for the prevention of fibrosis via the suppression of HSCs, a study of EVs from the chorionic plate-derived MSCs showed that these EVs suppressed the activation and proliferation of HSCs due to the presence of miR-125b in the cargo, thus preventing liver fibrosis [92]. Another study of EVs from ADSCs showed that the overexpression of miR-181-5p in the ADSC and their EVs cargos caused anti-fibrotic effects, with significant downregulation of collagen I, vimentin, α-SMA, and fibronectin [93]. Similarly, the ADSC EVs with a high level of miR-122 expression suppressed the activation and proliferation of HSCs via the reduction of insulin-like growth factor receptor 1 (*IGF1R*), Cyclin G(1) (*CCNG1*), and prolyl-4-hydroxylase α1 (*P4HA1*) gene expressions [94]. These ADSC EVs anti-fibrosis effects were replicated again in a mice model of NASH, in which the NASH mice with ADSC-EVs treatment had better liver improvements and greater anti-inflammatory macrophages [95]. However, no effect was observed in the lipid accumulation [95]. Similar anti-fibrosis effects on HSCs are shown in the other studies of EVs isolated from induced pluripotent stem cells (iPSC) [96], amnion-derived MSCs (AMSCs) [97], human liver stem cells (HLSCs) [98], and human umbilical cord-derived MSCs [99]. This evidence of hepatic protectiveness effects by the EVs is important, as the same effects were also seen before with MSCs (cells) treatment in various liver injury models due to bacterial lipopolysaccharide [100], thioacetamide (TAA) [101], ischemia/reperfusion [102], radiation [103], and D-galactosamine [104]. Since the hepatic protection effects are similarly seen with the treatment of the MSCs and their EVs, this MSC-EVs approach offers a significant advantage as it could eliminate the inefficient MSC cell transfers and rejection of the MSCs cell-based treatment [105].

The pre-clinical studies discussed above reported numerous advantages of using EVs as a treatment or therapy for liver diseases; however, no evidence is available for EVs application in the clinical trials [106]. A meta-analysis study investigated the application of MSCs as liver therapy in the clinical trials and reported that MSCs treatment, when compared to conventional treatment, is a relatively safe and significantly improved liver function [107]. However, most of these studies were still in phase I or II; thus, only short-term effects were available for the assessment [107,108]. Since EVs-based therapy (cell-free) may have more potential than the MSCs-based treatment, the lack of information on the clinical studies, standard EVs isolation protocol, effective doses, efficacy, cargo content, and the heterogeneous populations of the EVs make it challenging to conclude [108,109,110]. The heterogeneity nature of EVs causes different effects on their target cells; therefore, the studies focusing on the standard isolation of EVs to maintain the homogeneity of the EVs are important before the EVs could be used for an alternative therapy to improve liver condition in the future.

## 6. Conclusions

Based on these pre-clinical studies of EVs discussed above, the adipocyte-and hepatocyte-EVs could uncover the underlying molecular mechanism of NAFLD disease development and progression. Since the EVs carry bioactive components in their cargos, there is a possibility of a tightly regulated message encoded in the EVs. However, the available data are limited, and no definite markers of adipocyte-EVs or hepatocyte-EVs are confirmed yet. Thus, EVs studies are subjected to careful interpretation. Moreover, a gap exists in identifying what molecules are inside these EVs and how these molecules can interact with the recipient cells to modulate the metabolic changes. Understanding these molecular mechanisms will allow for the possible use of EVs as therapeutic tools. The application of using MSC EVs as a treatment option for liver or NAFLD is attractive, although currently, the evidence is limited. Further research to elucidate the content of these EVs and optimize the therapeutic efficacy is needed.

## Figures and Tables

**Table 1:** Summary of the reported extracellular vesicles (EVs) from various sources and their clinical importance related to non-alcoholic fatty liver disease (NAFLD).

Extracellular Vesicles	Cell Source	EVs-Derived Disease Model	Molecular Mediators in the EVs Cargos	Recipient Targets	Interaction	NAFLD Relevance	Reference
Exosome	Visceral adipose tissue (VAT)	Leptin-deficient (*ob*/*ob*) B6 mice, B6 mice fed high-fat diets	RBP4	Bone marrow-derived macrophages (BMDM)	Increased production of MCSF, IL-6, and TNF-α	Activation of BMDM macrophages induced insulin resistance	[34]
Exosome	VAT	Human, Females with BMI > 30 kg/m^2^	MicroRNAs	TGF-β and Wnt/β-catenin signaling	TGF-β signaling and Wnt/β-catenin signaling among the top significant pathways	MicroRNAs in the exosomes derived from the obese visceral adipocytes are predicted to regulate inflammatory and fibrotic signaling pathways	[36]
Exosomes	VAT	Human, Females with BMI 35–46 (obese)	-	Hepatocytes and Hepatic stellate cells (HSCs)	Induced the expressions of *TIMP-1*, *TIMP-4*, *SMAD-3*, *MMP-9*, *integrins ανβ-5* and -*8*	Dysfunctional ECM regulation in the liver cells due to obese adipocyte exosomes	[37]
Exosome	Adipose tissue macrophages (ATM)	C57BL6 mice fed high-fat diets (in vivo), 3T3-L1 adipocytes (in vitro)	MicroRNAs (specifically miR-155)	L6 muscle cells and primary hepatocytes	Enriched miR-155 in the obese ATM-derived exosomes suppressed the expression of its target gene, *PPARγ*, and the downstream pathways	MicroRNAs cargos of secreted ATM-derived exosomes induced insulin resistance and glucose intolerance	[38]
Exosome	ATM	C57/BL6 mice fed high-fat diets	MicroRNAs (specifically miR-29a)	PPARD	MiR-29a interacts with PPARD to promote obesity-induced insulin resistance	ATM-derived exosomal miR-29a impairs insulin sensitivity in vitro and in vivo	[39]
Exosome	Adipose tissue	C57BL/6J (B6) mice fed high-fat diets and B6 *ob*/*ob* mice	miR-141-3p	AML12 liver cells	Decreased miR-141-3p expression caused impaired insulin signaling and glucose uptake in the hepatocytes	Exosomes from obese adipose tissues induced hepatocyte insulin resistance	[40]
Exosomes	Adipocytes	Human, Females with BMI 51.2±8.8 kg/m^2^	MicroRNAs	Insulin receptor signaling pathway	Circulating adipocyte-derived exosomes are modified following gastric bypass surgery and correlated with improved post-surgery insulin sensitivity	Bypass surgery intervention changed the properties of the exosomes derived from the adipocyte tissues	[41]
Exosomes	Hepatocytes	C57BL/6 mice fed high-fat diets	Sphingosine-1-phosphate (S1P)	BMDM	Hepatocytes EVs with S1P-enriched activated macrophage chemotaxis via the S1P1 receptor	Lipotoxic hepatocytes-derived EVs induce macrophage chemotaxis	[48]
Exosomes	Hepatocytes	C56Bl/6J mice fed high-fat diets	Pro-inflammatory lipids (C16:0 ceramide)	Macrophages	Lipotoxic hepatocyte-EVs stimulated macrophage chemotaxis via S1P generation	Lipotoxic hepatocytes-derived EVs induce macrophage chemotaxis	[49]
Exosomes	Hepatocytes	C56Bl/6J mice fed high-fat diets	miR-130a-3p	Adipocytes, PHLPP2	High expression of miR-130a-3p suppressed *PHLPP2* expression to activate AKT-AS160–GLUT4 signaling pathway in adipocytes	miR-130a-3p regulates glucose metabolism by increasing glucose uptake	[50]
Exosomes	Hepatocytes	Huh7 cells treated with palmitate	MicroRNAs (especially miR-122 and miR-192)	HSCs	Hepatocyte-EVs increased the expression of pro-fibrotic markers such as *α-SMA*, *TGF-β*, and *COL1A1* in HSCs.	Activation of fibrosis molecules	[51]
Microvesicle	Hepatocytes	HepG2 cells treated with palmitate	-	HSCs and hepatocytes	Lipotoxic hepatocyte-microvesicle internalization activated NLRP3 inflammasome via NF-kB, pro-caspase-1 and pro-interleukin-1, IL-1β	Activation of inflammatory phenotype in macrophages	[52]
Extracellular vesicles	Adipocytes	Patients with vascular disease	Cystatin-C	Monocytes, endothelial cells, platelets	The elevated level of EVs-cystatin C associated with metabolic complications of obesity	Low HDL cholesterol was significantly related to higher EV-cystatin C levels	[42]
Extracellular vesicles	Hepatocytes	C57BL/6 mice with choline-deficient amino acid diet	MicroRNAs (especially miR-128-3p)	HSCs	miR-128-3p suppressed the expression of *PPARγ* in HSCs	Activation of the HSCs	[53]
Extracellular vesicles	Hepatocytes	C57BL/6 mice model of NASH	TRAIL	IL-1β and IL6 in BMDM	Lipotoxic hepatocytes induced releases of pro-inflammatory EVs that activated macrophage via the death receptor 5 (DR5)-dependent manner	Activation of inflammatory phenotype in macrophages due to excess lipids in the liver cells	[11]
Extracellular vesicles	Hepatocytes	Primary hepatocytes and Huh7 cells treated with palmitate	CXCL10	BMDM	Lipotoxic EVs have enriched of CXCL10, a chemotaxis inducer for macrophages	Lipotoxic hepatocytes-EVs activated macrophage chemotaxis	[54]
Extracellular vesicles	Hepatocytes, macrophage, neutrophil, platelet	C56BL/6J mice fed high-fat diets	-	Changes in liver condition (onset of NASH)	Quantitative evolution of hepatocyte-, macrophage- and neutrophil-derived EVs correlated well with the histology of NASH	Circulating EVs derived from different cells are enriched at a specific time, according to NASH development	[45]
Extracellular vesicles	Serum	C56BL/6J mice fed high-fat diets and underwent aerobic training	MicroRNAs (especially miR-122, miR-192, and miR-22)	Hepatocytes, adipocytes	Serum EVs miR-22 expression was associated with adipogenesis and insulin sensitivity markers in adipocytes. Liver *PPARγ* expression was negatively correlated with serum miR-122 level	Aerobic training prevented obesity-induced steatohepatitis	[43]
Extracellular vesicles	Plasma, hepatocytes	C56BL/6J male mice fed high-fat diets	S1P	BMDM and HSCs	Circulating EVs were enriched in mice with high-fat diets	Activation of inflammatory phenotype in macrophages	[55]
Extracellular vesicles	Hepatocytes	C57BL/6J mice fed high-fat diets	MicroRNAs (especially miR-122, let-7e-5p, miR-31-5p and miR-210-3p)	Adipocytes	Increased miR-122, let-7e-5p, miR-31-5p and miR-210-3p expression in adipocytes	Hepatocyte-EVs increased fat accumulation and the expression of lipogenesis genes	[56]
Extracellular vesicles	Hepatocytes	HepG2 cells treated with cobalt chloride (CoCl_2_) or excess fatty acids	-	HSCs	Hepatocyte-EVs increased the expression of the pro-fibrotic markers of *TGFβ-1*, *CTGF*, *COL1A1,* and *α-SMA* in HSCs	Activation of the fibrosis and HSCs	[57]
Extracellular vesicles	Hepatocytes	HepG2 cells treated with cobalt chloride (CoCl_2_) or excess fatty acids	-	Kupffer cells	Hepatocyte-EVs have enrichment of the pro-inflammatory cytokines and inflammasomes (interleukin-1β, NLRP3, and ASC). Hepatocyte-EVs induced chemotaxis in Kupffer cells	Lipotoxic hepatocytes-EVs activated Kupffer cells chemotaxis	[58]
Extracellular vesicles	Hepatocytes	Hepatocytes treated with palmitate	MicroRNAs (especially miR-1)	Human umbilical vein endothelial cells (HUVECs)	miR-1 suppressed expression of *KLF-4* and increased the NF-κB activity	Hepatocyte-EVs induced endothelial cell inflammation	[59]

Abbreviation: Adipose tissue macrophages (ATMs), Alpha-smooth muscle actin (α-SMA), Apoptosis-associated speck like protein containing a caspase recruitment domain (ASC), Body Mass Index (BMI), Bone marrow–derived macrophages (BMDM), Connective tissue growth factor (CTGF), C-X-C-motif chemokine 10 (CXCL10), Extracellular matrix (ECM), Extracellular vesicles (EVs), Geranylgeranyl diphosphate synthase (Ggpps), Human umbilical vein endothelial cells (HUVECs), Interleukin-6 (IL-6), Kruppel-like factor 4 (KLF4), Macrophage colony-stimulating factor (MCSF), Matrix metalloproteinase-9 (MMP-9), Non-alcoholic steatohepatitis (NASH), NLR family pyrin domain containing 3 (NLRP3), Nuclear factor kappa B (NF-κB), Peroxisome proliferator-activated receptor delta (PPARD), Peroxisome proliferator-activated receptor gamma (PPARγ), PH Domain And Leucine Rich Repeat Protein Phosphatase 2 (PHLPP2), Retinol binding protein 4 (RBP4), Sphingosine-1-phosphate (S1P), Tissue inhibitor of matrix metalloproteinase-1 (TIMP-1), Tissue inhibitor of matrix metalloproteinase-4 (TIMP-4), TNF-related apoptosis-inducing ligand (TRAIL), Transforming growth factor beta (TGF-β), Tumor necrosis factor-alpha (TNF-α), Visceral adipose tissue (VAT).

**Table 2:** Comparisons of extracellular vesicles (EVs) treatment from various mesenchymal stem cells and their clinical relevance to liver injury and disease.

Extracellular Vesicles	Cell Source	Molecular Mediators in the EVs Cargos	Recipient Targets Model	Interaction	Clinical Relevance	Reference
Exosomes	Human umbilical cord MSCs (hucMSC)	mRNA, surface adhesion molecules	Acute liver injury mice model (CCl4 treatment)	hucMSC exosomes recovered AST activity, reduced *COL1A1*, *COL3A1*, and *TGF-β1* expressions	Alleviation of liver fibrosis	[73]
Exosomes	hucMSC	GPX1	Acute liver injury mice model (CCl4 treatment)	Reduction of hepatic ROS and apoptosis by increasing the ERK1/2 and BCL-2 and decreasing the IKKB/NFkB/Casp-9/-3 pathway	The recovery of hepatic oxidant injury	[81]
Exosomes	hucMSC	-	Acute liver injury mice model (LPS and D-galactosamine treatment), RAW264.7 macrophages	Reduction of *NLRP3*, *Casp-1*, *IL-1β*, *IL-6* expressions in the macrophage, liver ALT and AST levels, and the restoration of damaged liver tissue	Reduced inflammation and liver damage is repaired	[83]
Exosome	Chorionic plate-derived MSCs (CP-MSCs)	miR-125b	Acute liver injury mice model (CCl4 treatment), hepatic stellate cells (HSCs)	miR-125b suppressed the activation of Hh signaling that promotes fibrosis	Suppression of the HSCs activation and proliferation	[92]
Exosomes	MSCs	-	Acute liver injury mice model (CCl4 treatment), hepatocytes	MSCs exosomes activated proliferation genes and prevented apoptosis	MSC-derived exosomes have hepatoprotective effects against acute-liver injury	[74]
Exosomes	Adipose tissue-derived MSCs (AMSCs)	miR-17	Acute liver injury mice model (LPS and D-galactosamine treatment), Kupffer cells	miR-17 reduced *TXNIP* expression and suppressed the NLRP3 inflammasome activation in Kupffer cells	Reduction of inflammatory activation in Kupffer cells	[89]
Exosomes	AMSCs	miR-181-5p	Acute liver injury mice model (CCl4 treatment), HSCs	miR-181-5p increased autophagy and reduced liver fibrosis by inhibiting the STAT3/BCL-2/Beclin-1 pathway HSCs *COL1A1*, *VIMENTIN*, *α-SMA*, and *FN1* expressions were reduced	AMSCs exosomal miR-181-5p has an anti-fibrotic role	[93]
Exosomes	AMSCs	miR-122	Acute liver injury mice model (CCl4 treatment), HSCs	miR-122 reduced the expression of *IGF1R*, *CCNG1*, and *P4HA1* in HSCs	Suppression of the HSCs proliferation and collagen maturation	[94]
Exosome	Adipose-derived stem cells (ADSC)	STAT3	Mice fed high-fat diets, macrophages	ADSC exosomes improved insulin sensitivity, reduced obesity, and alleviated hepatic steatosis, by inducing the anti-inflammatory phenotypes in M2 macrophages via the transactivation of arginase-1 by exosome-STAT3	Improvement of insulin regulation and hepatic steatosis	[72]
Exosomes	Bone-marrow-derived MSC (BMSCs)	-	Acute liver injury mice model (CCl4 treatment), hepatocytes (Acetaminophen or hydrogen peroxide treatment)	Reduced ROS production and prevented oxidative stress, as well as improved liver regeneration and recovery	The recovery of hepatic oxidant injury	[76]
Exosomes	BMSCs	-	Hepatocytes (LPS and D-galactosamine treatment)	BMSCs exosomes reduced the pro-apoptotic proteins BAX, and cleaved Casp-3, and increased the expression of the anti-apoptotic *BCL-2*	Induce autophagy and protect hepatic cells from damage caused by various stresses by mediating autophagy	[80]
Exosome	BMSCs	-	Acute liver injury mice model (CCl4 treatment), HSCs	BMSCs exosomes alleviated liver fibrosis and inflammation, as well as reduced the expression of Wnt/β-catenin pathway components (*PPARγ*, *Wnt3a*, *Wnt10b, β-catenin*, *WISP1*, *CCND1*, *α-SMA*, and *COL1A1*) in HSCs and liver tissue	Alleviation of liver fibrosis via the inhibition of Wnt/β-catenin signaling	[79]
Exosomes	Human-induced pluripotent stem cell-derived mesenchymal stromal cells (hiPSC-MSCs)	-	Liver injury mice model (ischemia/reperfusion surgery), hepatocytes	hiPSC-MSCs exosomes reduced AST and ALT levels and increased primary hepatocyte proliferation and synthesis of S1P	Protection against hepatic ischemia/reperfusion injury	[86]
Exosomes	Human menstrual blood-derived stem cells (MenSCs)	ICAM-1, angiopoietin-2, Axl, angiogenin, IGFBP-6, osteoprotegerin, IL-6, and IL-8	Acute liver injury mice model (LPS and D-galactosamine treatment), AML12 macrophage cells	MenSCs exosomes improved liver function and inhibited apoptosis with a reduction of active Casp-3	Inhibition of cell apoptosis and enhanced survival	[87]
Microvesicles (MVs)	Human liver stem cells (HLSC)	mRNAs	Hepatocytes	HLSC MVs activated cell proliferation and liver regeneration	Liver regeneration	[84]
Extracellular vesicles	HLSC	ASS1 protein and mRNA	Hepatocytes derived from ASS1 deficient HLSC	HLSC EVs restored ASS1 activity and urea production	Restoration of ASS1 function in deficient cells	[85]
Extracellular vesicles	HLSC		NASH mice model (choline-deficient amino acid diet)	HLSC EVs reduced fibrosis and inflammation markers (α-SMA), COL1A1, TGF-β1, TNF-α, IL-1β, and LTBP1	Reduction of inflammation and fibrogenesis	[98]
Extracellular vesicles	hucMSC	MnSOD enzyme	Liver injury mice model (ischemia/reperfusion surgery)	hucMSC EVs reduced neutrophils infiltration and alleviated hepatic oxidative stress	Inhibition of the oxidative stress and neutrophil inflammatory response	[82]
Extracellular vesicles	hucMSC	-	Liver injury mice model (*S. japonicum* infection), HSCs	hUCMSC EVs ameliorated liver injury and reduced the expression of *α-SMA*, *COL1A1*, and *COL3A1*, as well as HSCs proliferation	Suppression of HSCs proliferation and improved liver condition	[99]
Extracellular vesicles	Amnion-derived mesenchymal stem	-	NASH mice model (high-fat diets), Acute liver injury mice model (CCl4 treatment), HSCs and Kupffer cells	AMSC EVs reduced the expression of pro-inflammatory cytokines (TNF-α, IL-1β, IL-6, and TGF-β), fibrosis, Kupffer cell numbers, and HSC activation	Reduction of inflammation and fibrogenesis	[97]
Extracellular vesicles	BMSCs	Y-RNA-1	Liver failure mice model (D-galactosamine/TNF-α treatment), hepatocytes	BMSCs EVs reduced hepatic injury and apoptosis	Protective effect against hepatocyte apoptosis	[75]
Extracellular vesicles	BMSCs	-	Liver injury mice model (ischemia/reperfusion surgery), hepatocytes	BMSCs EVs reduced tissue necrosis, apoptosis, serum ALT, and increased expression of *NLRP12* and *CXCL1*, as well as increased the expression of *IL-6*	Reduction of tissue necrosis, inflammation, and apoptosis	[77]
Extracellular vesicles	Human mesenchymal stromal cell (hMSCs)	-	Liver injury mice model (ischemia/reperfusion surgery)	hMSCs EVs reduced hepatic necrosis and inflammatory genes (*HMBG-1*, *ICAM-1*, *HO-1*, and *IL-1β*)	Reduction of tissue necrosis and inflammation	[78]
Extracellular vesicles	Human embryonic stem cell-derived mesenchymal stroma cells	-	Liver injury mice model (thioacetamide treatment)	EVs reduced fibrosis, apoptosis, and regenerated liver cells	Regeneration of liver	[64]
Extracellular vesicles	Human adipose-derived stem cells (hASCs)	lncRNA H19	Acute liver injury mice model (D-galactosamine treatment)	hASCs EVs reduced the expression of inflammatory mediators and chemotactic factors	Inhibition of the liver inflammation	[90]
Extracellular vesicles	hASCs	-	NASH mice model (high-fat diets) with acute liver injury (LPS treatment)	hASCs EVs reduced serum ALT levels and inflammatory markers and macrophages	Inhibition of the liver inflammation	[95]
Extracellular vesicles	Human induced pluripotent stem cell (iPSCs)	MicroRNAs (specifically miR-92a-3p)	HSCs	iPSCs EVs reduced pro-fibrogenic markers (α–SMA, COL1A1, FN1, and TIMP-1), and HSC proliferation	Inhibition of fibrosis and HSCs proliferation	[96]

Abbreviations: Adipose tissue-derived MSCs: (AMSCs), Alanine aminotransferase (ALT), Alpha-smooth muscle actin (α-SMA), Argininosuccinate synthase-1 (ASS1), Aspartate aminotransferase (AST), B-cell lymphoma 2 (BCL-2), BCL-2 Associated X-protein (BAX), Bone-marrow-derived MSCs (BMSCs), C-X-C motif chemokine ligand 1 (CXCL1), Carbon tetrachloride (CCl4), Caspase (Casp), Chorionic plate-derived MSCs (CP-MSCs), Collagen type I (COL1A1), Collagen type III (COL3A1), Cyclin D(1) (CCND1), Cyclin G(1) (CCNG1), Extracellular signal-regulated kinases 1/2 (ERK1/2), Extracellular vesicles (EVs), Fibronectin (FN1), Glutathione peroxidase1 (GPX1), Hedgehog (Hh), Hepatic stellate cells (HSCs), Heme oxygenase-1 (HO-1), High mobility group box 1 protein (HMBG-1), Human adipose-derived stem cells (hASCs), Human-induced pluripotent stem cell–derived mesenchymal stromal cells (hiPSC-MSCs), Human induced pluripotent stem cell (iPSCs), Human liver stem cells (HLSC), Human menstrual blood-derived stem cells (MenSCs), Human umbilical cord MSCs (hucMSC), IκB kinase (IKKB), Insulin-like growth factor-binding protein 6 (IGFBP-6), Insulin-like growth factor receptor 1 (IGF1R), Intercellular adhesion molecule 1 (ICAM-1), Interleukin-1 β (IL-1 β), Interleukin-6 (IL-6),), Interleukin-8 (IL-8), Latent-transforming growth factor beta-binding protein 1 (LTBP1). Lipopolysaccharides (LPS), Mesenchymal stem cells (MSCs), Messenger RNA (mRNA), Microvesicles (MVs), Mitochondria-located antioxidant enzyme, manganese superoxide dismutase (MnSOD), NLR family pyrin domain containing 3 (NLRP3), NLR family pyrin domain containing 12 (NLRP12), Nonalcoholic steatohepatitis (NASH), Noncoding RNA Y (Y-RNA-1), Nuclear Factor kappa-light-chain-enhancer of activated B cells (NFkB), Reactive oxygen species (ROS), Peroxisome proliferator-activated receptor gamma (PPARγ), Prolyl-4-hydroxylase α1 (P4HA1), Signal transducer and activator of transcription 3 (STAT3), Sphingosine-1-phosphate (S1P), Thioredoxin Interacting Protein (TXNIP), Tissue inhibitor of metalloproteinases–1 (TIMP-1), Transforming growth factor beta-1 (TGF-β1), Tumor necrosis factor-alpha (TNF-α), Wingless and Int-1 (Wnt), Wnt1-inducible signaling pathway protein-1 (WISP1).
